# In Vitro Antibacterial Activity of Raw Honey From *Apis mellifera*, Awi Zone, Amhara, Ethiopia

**DOI:** 10.1002/mbo3.70076

**Published:** 2025-10-17

**Authors:** Bulti Kumera, Bekele Gebreamanule, Belsti Atnkut, Mestawot Merid, Tadele Tilahun, Alemu Tsega, Amare Fassil, Yitayih Dessie, Banchalem Kassie

**Affiliations:** ^1^ Department of Biology, College of Natural and Computational Science Injibara University Injibara Ethiopia; ^2^ Doctoral School of Biology Hungarian University of Agricultural and Life Sciences Gödöllö Hungary; ^3^ Department of Chemistry, College of Natural and Computational Science Injibara University Injibara Ethiopia

**Keywords:** agar well diffusion assay, antibacterial activity, honey, pathogen

## Abstract

Honey has been used for the treatment of various illnesses since time immemorial. Honey is employed as a remedy for the treatment of burns, microbial infections, cardiovascular disorders, and gastrointestinal problems. The objectives of this study were to evaluate physicochemical characteristics and in vitro antibacterial properties of honey produced by *Apis mellifera* against *Staphylococcus aureus*, *Pseudomonas aeruginosa*, *Escherichia coli*, *Salmonella typhi* and *Klebsiella pneumoniae*. Honey samples were collected from three districts of the Awi zone from April to June 2023. The pH and moisture of collected honey were determined following the standard procedure. An antimicrobial susceptibility test of honey samples was performed using an agar well diffusion assay. The moisture contents and pH of collected honey samples from the three study sites were 13 ± 0.07–20 ± 0.09 and 3.76–4.01, respectively. There was no significant (*p* > 0.05) difference in pH between honey samples collected from the three sites. Similarly, there was no significant statistical association among the moisture content of honey samples (*p* > 0.05). Pure 100% honey samples showed the greatest inhibition zone against *E. coli* (19.20 mm) and the lowest against *P. aeruginosa* (2.4 mm). Honey concentration of 25% showed the lowest inhibition zone against all tested bacterial pathogens. Antibacterial effects of honey solutions decreased upon serial dilution from 100% to 25%. Our finding suggests that raw honey can be used as therapeutic agents against bacterial disease.

## Graphical abstract

Honey samples were collected from districts of Awi zone. The moisture contents and pH of honey samples were 13 ± 0.07–20 ± 0.09 and 3.76–4.01, respectively. Pure 100% honey samples showed the greatest inhibition zone with 25% showed the lowest inhibition zone against tested bacterial pathogens.

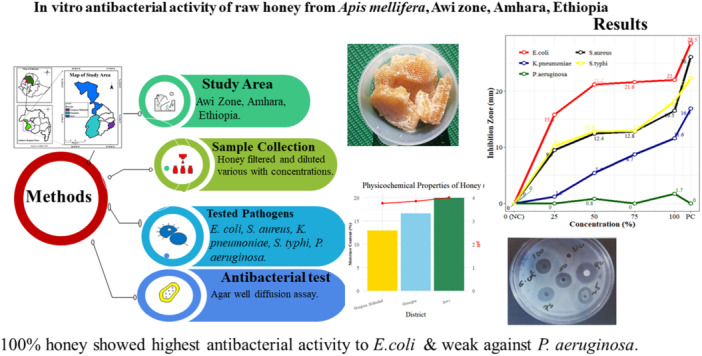

## 1 Introduction

Natural product utilization is gaining popularity as a method for food preservation and medical therapies (Albaridi 2019). Honey is a naturally occurring commodity made by honeybees, and 95%–97% of its dry weight is made up of carbohydrates (Bucekova et al. 2018). The primary components of all honey are the same, although the composition changes depending on the floral species that the bees feed on Israili (2014). Humans have been using honey to cure a wide range of illness for at least 2700 years (Lusby et al. 2005). While the ancient Greeks utilized honey as a treatment for gout, pain, fever, and wound healing, the Egyptians employed it as a topical ointment, a bandage for wounds, and to embalm their dead (Nolan et al. 2019). Bioactive substances from plants and bees are found in honey. These bioactive compounds are crucial in eliminating and preventing the spread of harmful microorganisms (Libonatti et al. 2014). Honey's ability to destroy microbes has been related to its high osmotic effect, high acidity, high hydrogen peroxide content, and botanical origin (Manyi‐Loh et al. 2010). The antibacterial activities of honey have been known since the 19th century (Kwakman and Zaat 2012).

Effective inhibitory activity of Manuka honey against human pathogenic bacteria, including *Escherichia coli*, *Enterobacter aerogenes*, *Salmonella typhimurium*, and *Staphylococcus aureus*, was reported (Sekar et al. 2023). Moreover, Scepankova et al. (2017) showed the antibacterial properties of various types of honey from different sources against resistant strains isolated from patients, including *Pseudomonas aeruginosa*, *E. coli*, *S. aureus*, *Staphylococcus epidermidis*, *S. typhimurium*, *Bacillus cereus*, *Bacillus subtilis*, and *Listeria monocytogenes*. Treatment of bacterial disease becomes challenging due to the development of bacterial resistance against antimicrobial drugs (Feás et al. 2013). In addition, the medical community has recently rediscovered the use of honey as a therapeutic ingredient, and it is becoming more widely accepted as an antibacterial treatment for bed sores, gastrointestinal ulcers, and other surface infections (Aurongzeb and Azim 2011). Approximately 80% of Ethiopian populations get their primary medical treatment via traditional methods. Honey is frequently used as a traditional treatment for gastrointestinal disorders, wound healing, diarrhea, and allergies (Kassaye et al. 2006). Therefore, investigating the physicochemical and antibacterial properties of honey is essential.

## 2 Materials and Methods

### 2.1 Study Site

The study was carried out at the Awi zone, Amhara Regional State, Ethiopia. It is situated at an elevation between 700 and 2920 above sea level and covers an area of land about 857,886 ha. The total area of the Awi zone is about 9148.43 km^2^ (Mekonnen 2018). The capital city of the zone was Injibara located about 447 km away from Addis Ababa and 118 km from Bahir Dar. According to 2007 central statistical agency (CSA) of Ethiopia, the Awi zone has a total population of 982,942 of whom 491,865 are men and 491,077 women. The Awi zone capital city Injibara was about 118 km from Bahir Dar and 447 km from Addis Ababa. The Awi zone has a total population of 982,942, of which 491,865 are men and 491,077 are women, according to the CSA of Ethiopia in 2007.

**Figure 1:**
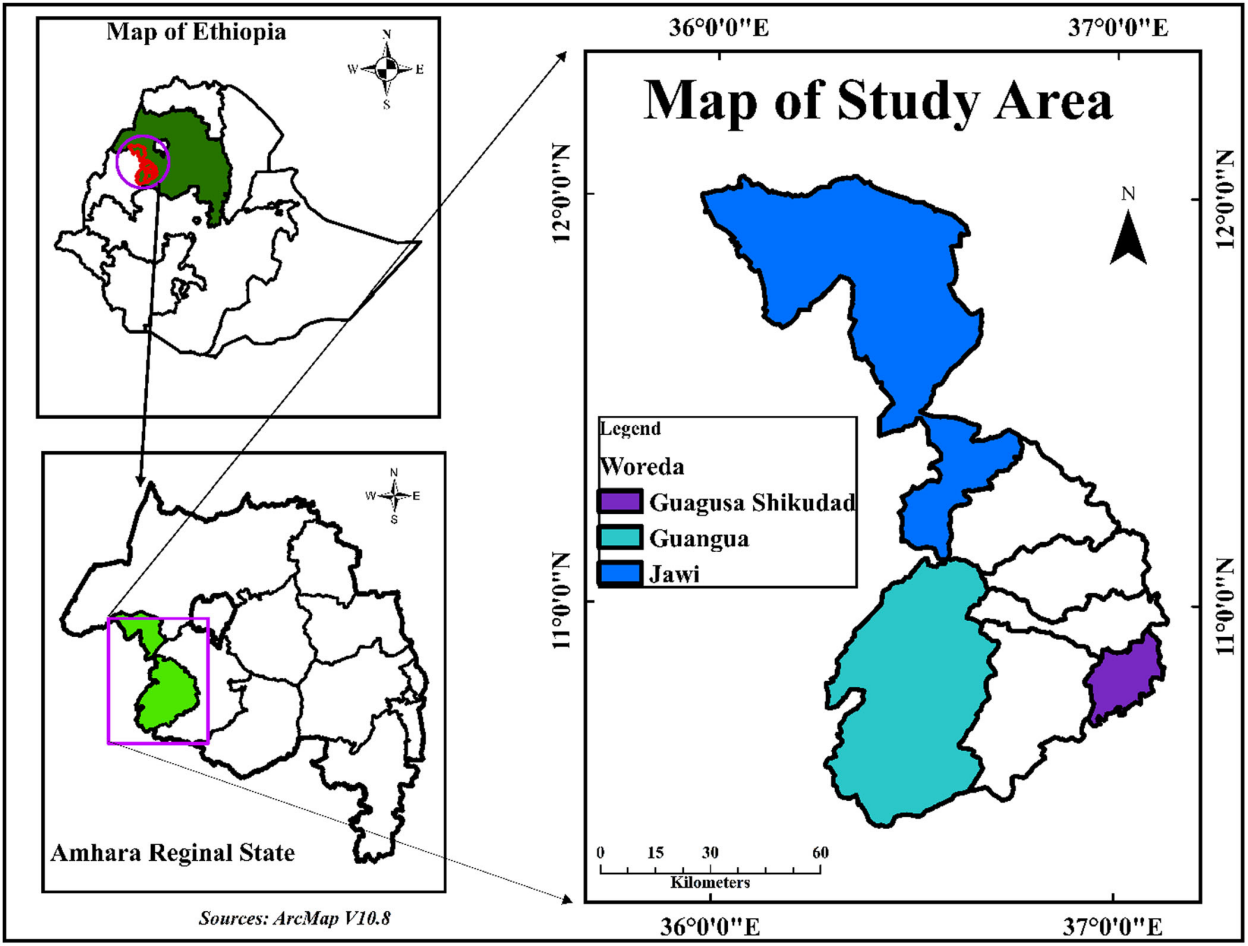
Map of study districts.

The locations of the sampling districts are depicted in Figure 1.

### 2.2 Honey Sample Collection Procedure

A total of 21 honey samples were collected from three districts of Awi zone, namely, Guagusa shikudad, Guangua, and Jawi, during the honey harvesting season, April–June 2023, using a sterilized screw caped sterile bottle. The samples were collected from local beekeepers during market days. Aseptically collected honey samples were brought to Injibara University, the biology department laboratory and filtered using sterile gauze under a biological safety cabinet. The streak plate method was employed to inoculate honey samples on sterile nutrient agar, followed by incubation at 37°C for 24 h to check purity. Honey samples without contamination were stored in a cool and dark place away from bright light.

### 2.3 Physicochemical Characteristics of Honey

#### 2.3.1 pH Measurement

The pH of honey samples was measured using a calibrated pH meter (Damto et al. 2023).

#### 2.3.2 Moisture Content

The moisture content of honey samples was determined using a formula developed by the Association of Official Analytical Chemists (Os et al. 2020). Moisture content (%) = {*M*_2_ − *M*_3_/*M*_2_ − *M*_1_} ∗ 100, where *M*_1_ is the weight of crucible (g), *M*_2_ the weight of crucible and sample (g), and *M*_3_ the crucible and dried sample weight (g).

### 2.4 Source of Test Organisms

Standard test tube slants of bacterial cultures, *E. coli* (ATCC 25922), *S. aureus* (ATCC 25923), *Salmonella typhi* (ATCC 14028), *P. aeruginosa* (ATCC 27853), and *Klebsiella pneumoniae* (ATCC 13883), were taken from Amhara Public Health Institute, Bahir Dar, Ethiopia. Test tube slant cultures were streaked on nutrient agar and incubated overnight at 35 ± 1°C for 24 h to check the viability of the cells at Injibara University. Viable bacterial cultures were preserved by streaking on sterile nutrient agar slant for further work.

### 2.5 Preparation of Honey Concentrations

Different dilutions of 25%, 50%, 75%, and 100% pure honey samples were prepared. A 75% honey concentration (v/v) was prepared by diluting 0.75 mL of honey with 0.25 mL of sterilized distilled water. Likewise, 0.5 mL of honey mixed with 0.5 mL of distilled sterile water for a 50% concentration and 0.25 mL of honey mixed with 0.75 mL of sterile distilled water for a 25% honey solution (v/v) (Luitel et al. 2023).

### 2.6 Antimicrobial Susceptibility Test of Honey

The antibacterial properties of honey samples were assessed using agar well diffusion techniques (Matzen et al. 2018). Pure bacterial isolates were taken from a nutrient agar slant and subcultured on nutrient agar, then incubated at 37°C for 24 h. About 3–5 isolated colonies of bacterium were inoculated into sterile nutrient broth and mixed thoroughly. Following incubation at 37°C for 6 h, the culture was compared with 0.5 MacFarland standards. The broth culture was evenly distributed on sterile Mueller Hinton agar medium using a sterile cotton swab. To absorb additional liquids, the plates were placed on a bench. Wells in culture medium were prepared using a sterile 6‐mm cork borer. Honey concentrations of 100%, 75%, 50%, and 25% were added to the plate wells using a micropipette. The plates were incubated at 37°C at 24 h (Mama et al. 2019). The diameter inhibition zones were recorded using a ruler (mm) and the results were recorded. Sterile distilled water was used as a negative control, whereas a chloramphenicol antibacterial disk was used as a positive control. Inoculated plates of the experiment were replicated three times for each standard bacterial species.

### 2.7 Statistical Analysis

SPSS version 16.0 was used for data analysis. Mean ± standard deviations (SD) of three measurements were used to express the experiment results. Analysis of variance (ANOVA) was employed to determine the significance difference among variables (*p* < 0.05).

## 3 Results and Discussions

### 3.1 Physicochemical Properties of Honey (pH and Moisture Content)

The present finding revealed that the mean moisture content (%) and pH of collected honey samples from the three districts (Guagusa Shikudad, Guangua, and Jawi) were found to be 13% ± 0.07%–20% ± 0.09% and 3.76 ± 0.16–4.01 ± 0.24, respectively. One‐way ANOVA revealed the absence of a significant statistical association between the pH of honey samples collected from the three districts (*p* > 0.05). Similarly, there was no significant statistical association among the moisture contents of honey samples collected from selected districts (*p* > 0.05). The moisture content of honey predominantly affects its quality, stability, and resistance to spoilage by yeast fermentation (Kumari et al. 2021).

The moisture content of honey can naturally vary from 13% to 23% depending on the source of honey, climatic conditions and other factors (Sintayehu et al. 2022). The average moisture content of investigated honey samples ranged from 13% ± 0.07% to 20% ± 0.09% with a mean value of 16.66% ± 0.12%. Nearly in agreement with our finding, Lewoyehu and Amare (2019) reported that the moisture composition of laboratory analyzed honey was ranged from 16.34% ± 0.26% to 19.83% ± 0.43%. Almost similar finding was reported by Gregorio et al. (2020), who revealed that the moisture contents of the tested honey samples range between 17.78% and 18.51%. Berhe et al. (2018) also reported nearly similar moisture content of honey samples with a mean value of 18.76% ± 1.09%. Moreover, Gebeyehu and Jalata (2023) found a mean moisture content of honey samples 19.82% ± 0.22%. In contrast to our results, a higher honey moisture content of 16.9%–32.4% was reported by Brown et al. (2020). In addition, Nweze et al. (2017) found a honey with a lower mean moisture content of 11.74% ± 0.47%. The maximum moisture content of raw honey was not higher than 23%, as per Codex Alimentarius 2001 (Veggeland and Borgen 2005), and our result was found within this standard. The texture, stability, and shelf life of honey were affected by its pH, which was crucial for both extraction and storage (Thakur et al. 2022).

The physicochemical analysis of honey samples in our investigation confirmed acidic pH ranging from 3.76 ± 0.16 to 4.01 ± 0.24 with a mean value of 3.85 ± 0.25. Organic acids, including gluconic, pyruvic, malic, and citric acids, are thought to be responsible for the low pH of honey (Bogdanov et al. 2004). The floristic composition and floral diversity of the environment are causes for the variation in pH between various honey samples (Fahim et al. 2014). But our findings revealed the absence of statistical significance between pH and honey samples from the three districts (*p* > 0.05). Comparable with our results (Fahim et al. 2014) reported that the pH of tested honey samples remained between 3.14 and 4.19. Similarly, the pH of honey samples collected from west Shewa districts of Ethiopia was determined to be 3.77–4.22 (Mulugeta 2017). Also, a mean 3.59 ± 0.25 pH measurement was reported from eastern Amhara, Ethiopia (Melaku and Tefera 2022). Furthermore, similar findings were reported in Gamozone, Southern Ethiopia, showing a mean honey pH of 3.80, which is in agreement with our results (Yemane et al. 2022). The low pH of honey has the advantage to prevent the growth of microorganism and indicates the honey samples freshness (Reshma et al. 2016).

### 3.2 Antibacterial Activity of Honey

The findings of laboratory results indicated that the inhibition zone of bacterial growth pattern decreases from undiluted honey to the minimum dilution (Table 1). A one‐way ANOVA analysis revealed that the mean zone of inhibition produced by four different concentrations of honey showed a statistically significant difference against positive and negative controls (*p* < 0.05). At 100% concentration, all honey samples exhibited antibacterial properties. The inhibition zones of bacteria increased with increasing honey concentration. However, the 25% honey concentration showed the lowest bacterial growth inhibition zone against all tested bacteria. Moreover, the negative control (0%) sterile water did not show any inhibitory effect against the tested bacteria. The minimum zones of inhibition were measured to be 0 mm against *P. aeruginosa* from a 25% honey concentration. The maximum zone of inhibition was recorded at 100% honey concentration against *E. coli* (22.02 mm). The positive control chloramphenicol (30 µg) showed various levels of growth inhibition against all bacteria except *P. aeruginosa*.

**Table 1:** Antibacterial activity of honey at different concentrations against pathogenic bacteria.

Concentration (%)	*Escherichia coli*	*Staphylococcus aureus*	*Klebsiella pneumoniae*	*Pseudomonas aeruginosa*	*Salmonella typhi*
0 (NC)	0 ± 0^a^	0 ± 0^a^	0 ± 0^a^	0 ± 0^a^	0 ± 0^a^
25	15.73 ± 4.39^b^	9.21 ± 5.65^b^	1.23 ± 2.71^b^	0 ± 0^b^	10.08 ± 3.38^b^
50	21.18 ± 13.23^c^	12.42 ± 1.71^b^	5.43 ± 4.44^c^	0.57 ± 1.52^b^	12.63 ± 2.56^b^
75	21.68 ± 13.44^c^	13.26 ± 2.15^c^	8.71 ± 2.76^d^	0.62 ± 1.54^b^	12.76 ± 3.95^c^
100	22.02 ± 13.44^c^	16.45 ± 5.07^d^	11.55 ± 2.59^d^	1.93 ± 3.22^b^	18.16 ± 4.06^d^
PC	28.54 ± 23.56^d^	26.07 ± 45.03^e^	16.79 ± 67.24^e^	0 ± 0^c^	22.28 ± 32.90^e^

*Note:* Chloramphenicol (30 µg). Values were designated as mean ± SD of three replications. Mean values with different superscripts down the column indicate statistically significant differences at (*p* < 0.05).

Abbreviations: NC = negative control, sterile water, PC = positive control.

The majority of human pathogenic bacteria, including *S. aureus*, *P. aeruginosa*, *E. coli*, and *Streptococcus pyogenes*, was reported to be susceptible to honey (Visavadia et al. 2008). The highest zone of inhibition was recorded about 28.54 mm against *E. coli*, but no inhibitory effect was examined for *P. aeruginosa* (0 mm). A 100% honey concentration showed that a clear sequence of growth inhibition with *E. coli* exhibiting the greatest average inhibition zone of 22.02 mm, followed by *S. typhi* (18.16 mm) and *S. aureus* (16.45 mm). In contrast, *K. pneumoniae* and *P. aeruginosa* exhibited smaller inhibition zones of 11.05 and 1.93 mm. Moreover, 25% of honey concentration showed a clear zone of inhibitions 15.73, 9.2, and 10.08 mm for *E. coli, S. aureus*, and *S. typhi*, respectively. *P. aeruginosa* was not affected by 25% honey concentration, while *K. pneumoniae* growth inhibition was measured to be 1.23 mm. In agreement with our experimental findings, Rahman et al. (2011) noted that 100% honey concentration exhibited greater zones of inhibition than all lower concentrations, while a dilution of 6.25% showed no impact when tested against *S. aureus*, *E. coli*, *S. typhi*, *P. aeruginosa*, and *Shigella* species (Al‐Naama 2009) found that honey exhibited an inhibitory effect in vitro at concentrations of 100%, 75%, and 50% but not 25% when tested against *S. aureus, E. coli*, and *P. aeruginosa* with maximum inhibition zone was measured in 100% honey concentration (23 mm). Comparable with our finding (Mudenda et al. 2023) showed that 100% concentration of honey inhibits the growth of *S. aureus* and *E. coli* at 22 and 20 mm, respectively, whereas 25% growth inhibition was 15 and 13 mm, respectively. A report from Mustafa et al. (2022) also revealed that the highest mean zone of inhibition in *E. coli* was 37.5 mm at 100% concentration of the honey, whereas the lowest inhibition was observed against *Enterococcus faecalis* (23 mm). However, a lower inhibition zone of honey against bacteria was reported by Ndife et al. (2014), with *S. aureus* (1.70–5.85 mm), *E. coli* (2.10–6.10 mm), and *B. subtilis* (2.05–3.20 mm) for honey collected from various floral regions.

The inhibitory activity of honey against bacteria is due to osmotic effect, low pH, and production of hydrogen peroxide (Brudzynski 2020). *P. aeruginosa* was the most resistant Gram‐negative bacterium in our experimental studies for both honey concentration and positive control (Chloramphenicol). Comparable with our result, the inhibitory activity of honey was also minimal against clinical *P. aeruginosa* (Tesfaye et al. 2022). The resistance of *P. aeruginosa* to honey may result from mutations in chromosomal genes that control resistance genes, limited permeability of the cell wall, and genetic potential to express resistant mechanisms (Wilkinson and Cavanagh 2005).

## 4 Conclusions

The finding revealed that the moisture content and pH of the investigated honey samples were found to be within the acceptable limit of national and international standards. Among the five tested bacterial pathogens *E. coli* displayed the highest susceptibility towards honey concentrations; however, *P. aeruginosa* exhibited the highest level of resistance. The overall inhibition zone of honey against bacterial pathogens reduced upon dilution from 100% to 25%. Any significant growth inhibition against the tested bacteria was not recorded in a 25% honey concentration. Our result will be helpful for the utilization of honey as a therapeutic agent for the treatment of diseases caused by bacterial pathogens.

## Author Contributions

**Bulti Kumera:** conceptualization, methodology, writing – original draft, writing – reviewing and editing. **Bekele Gebreamanule:** methodology, data curation, software. **Belsti Atnkut:** formal analysis, investigation, writing original draft. **Mestawot Merid:** project administration, validation, visualization. **Tadele Tilahun:** investigation, formal analysis, resource. **Alemu Tsega:** software, supervision, validation. **Amare Fassil:** supervision, project administration, writing – review and editing. **Yitayih Dessie:** data curation, methodology, writing – original draft. **Banchalem Kassie:** supervision, resource, writing – review and editing. All authors read and approved the final manuscript.

## Ethics Statement

The authors have nothing to report.

## Conflicts of Interest

The authors declare no conflicts of interest.

## Supporting information


**Figure 1:** Honey sample taken from beekepeers. **Figure 2:** Honey sample filtration. **Figure 3:** Honey samples for LAB analysis. **Figure 4:** Honey sample pH measurement. **Figure 5:** Test tube slants of bacterial pathogen. **Figure 6:** Different honey concentration inoculated on Mueller Hinton agar. **Figure 7:** Inhibition pattern of honey concentration and sterile water (NC) and PC (Chloramphenicol) against *E. coli*. **Figure 8:** Antibacterial activity of honey against *P. aeruginosa* (No inhibition zone).

## Data Availability

The data that support the findings of this study are available in the supporting material of this article.
