# Supplementary material for: In Vitro Antibacterial Activity of Raw Honey From Apis mellifera, Awi Zone, Amhara, Ethiopia
Source: Microbiologyopen. 2025 Oct 17;14(5):e70076. doi: 10.1002/mbo3.70076 (PMC12532491; doi:10.1002/mbo3.70076)

- **Photography support of paractical work**

- **Figure S1: Honey sample taken from beekepeers**

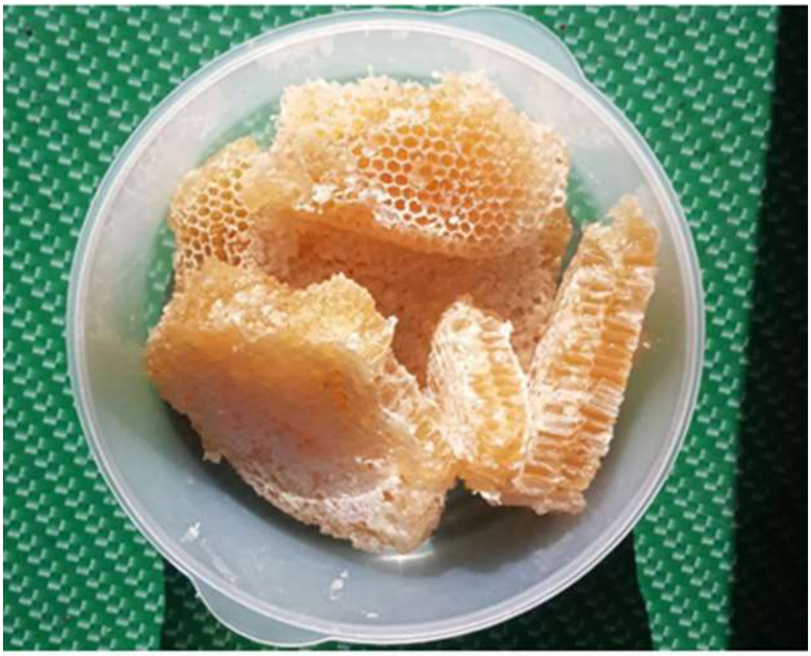

- **Figure S2: Honey sample filtration**

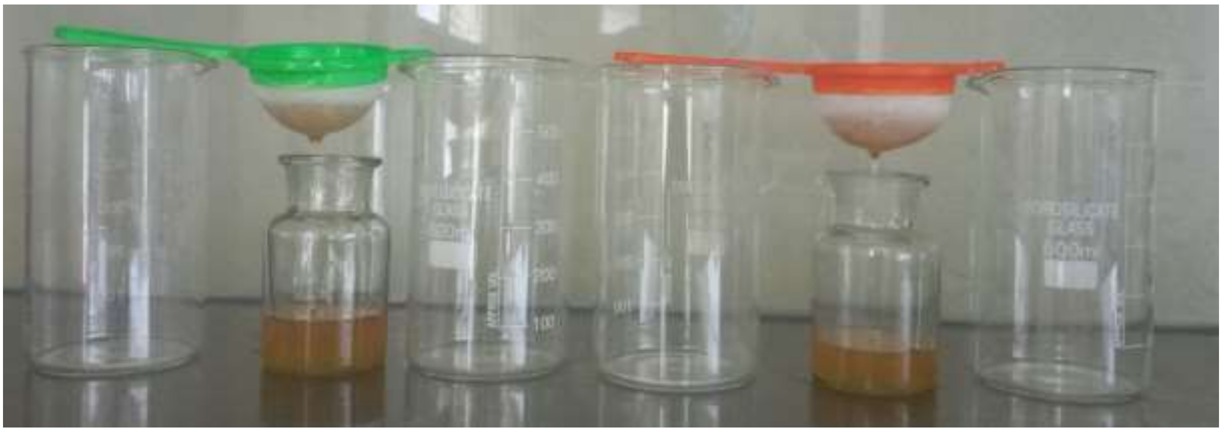

- **Figure S3: Honey samples for LAB analysis**

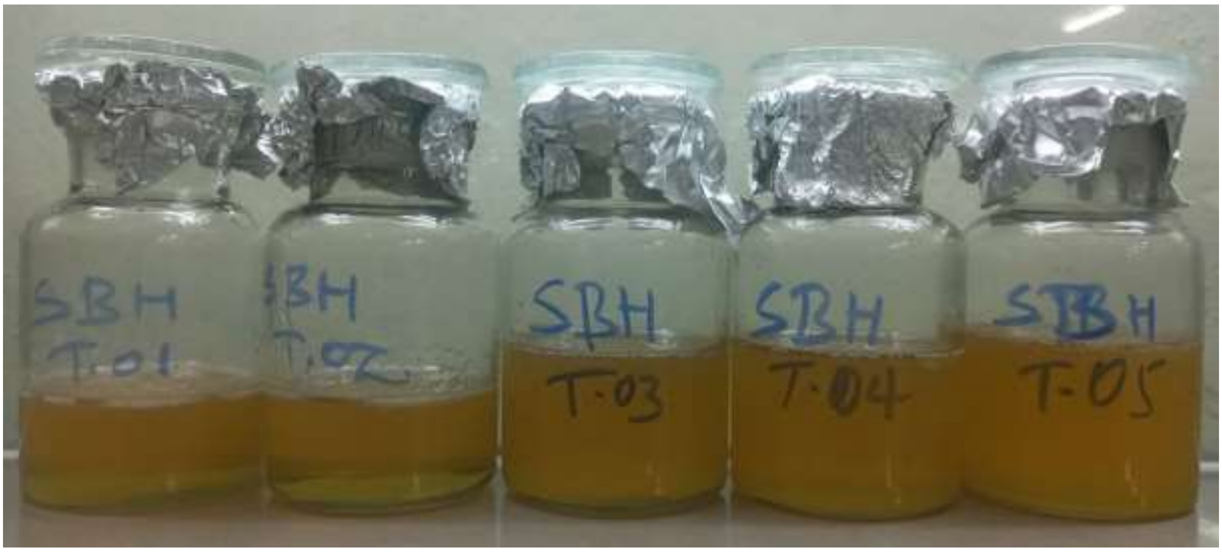

- **Figure S4: Honey sample pH measurement**

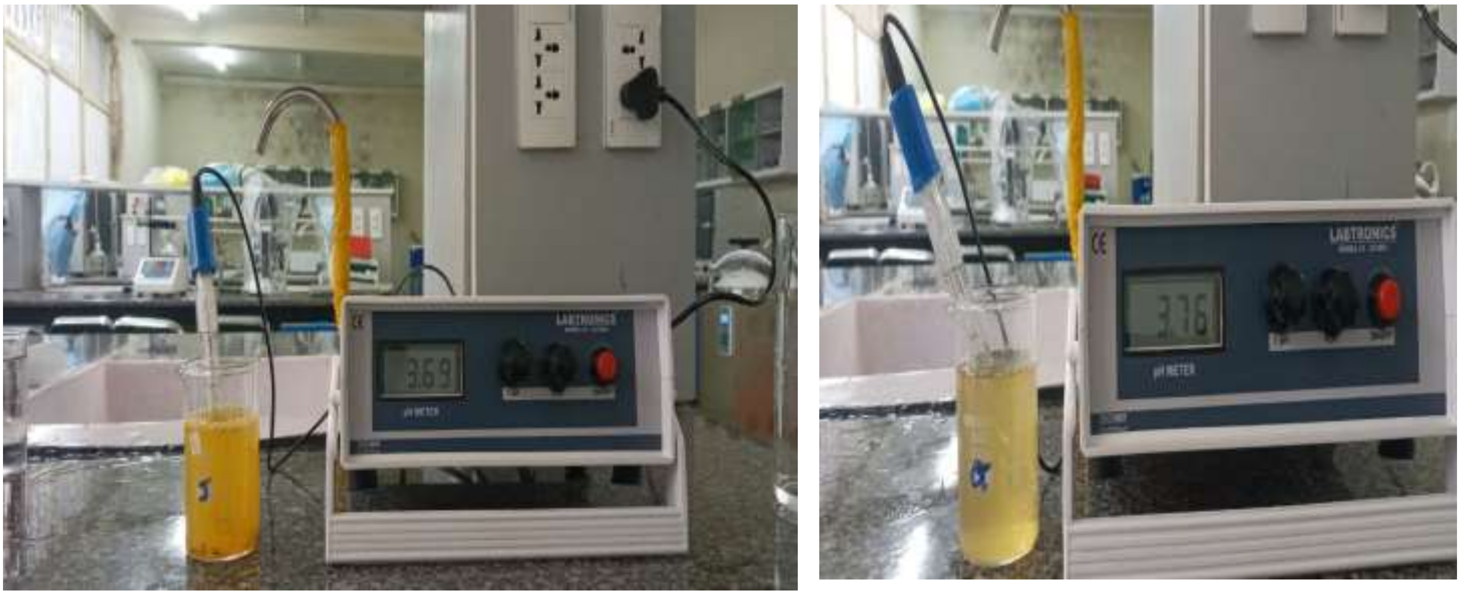

- **Figure S5: Test tube slants of bacterial pathogen**

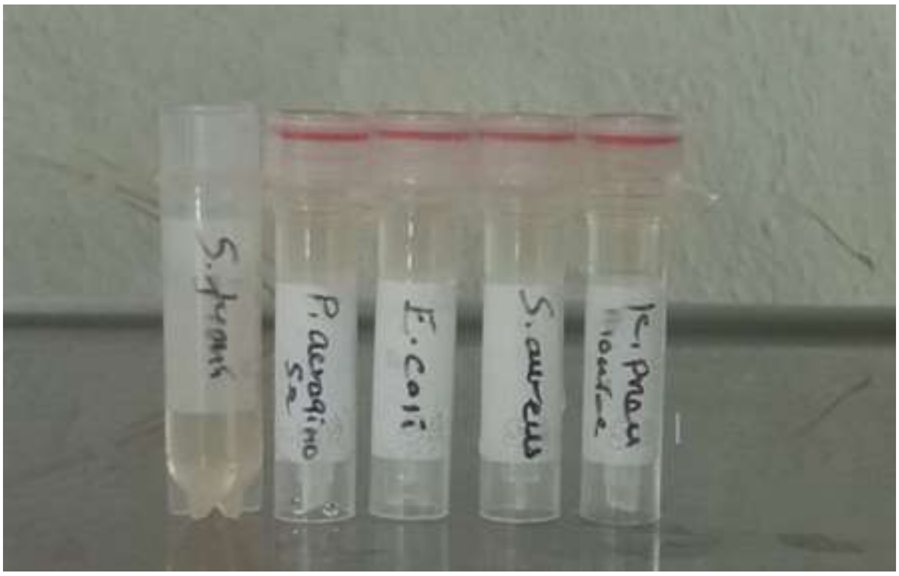

- **Figure S6: Different honey concentration inoculated on Mueuller hinton agar**

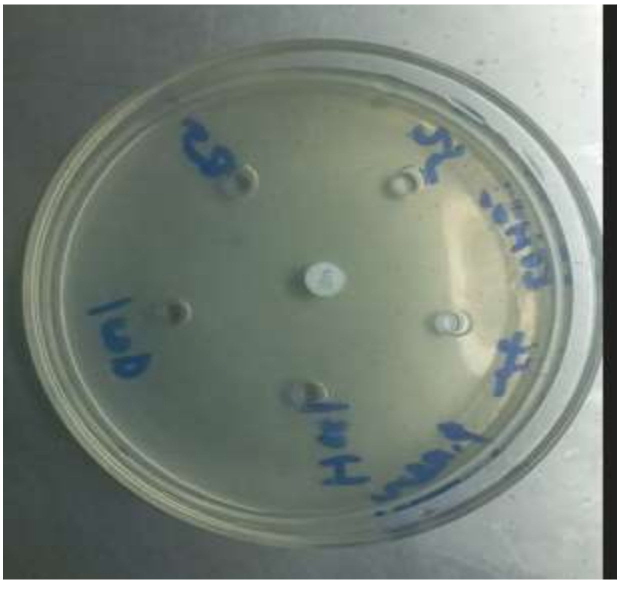

Figure S7: Inhibition pattern of honey concentration and sterile water (NC) and PC(Cloramphenicol) against *E.coli*

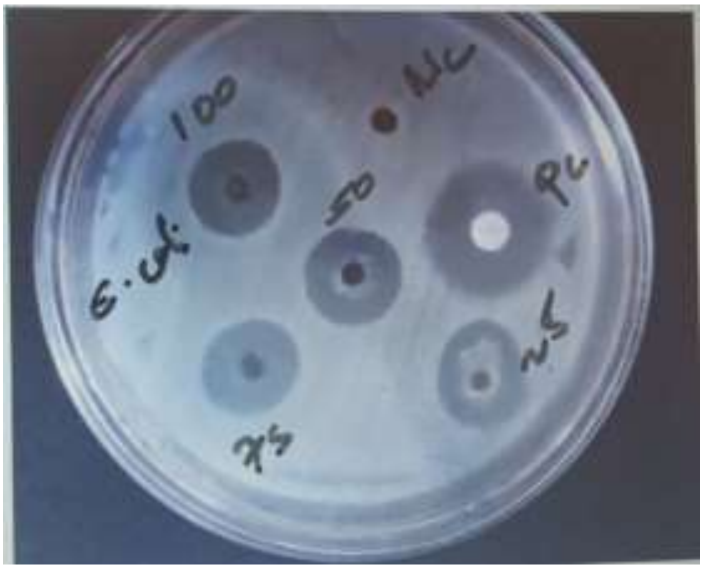

Figure S8: Antibacterial acitivity of honey against *P.aeruginosa* (No inhibition zone)

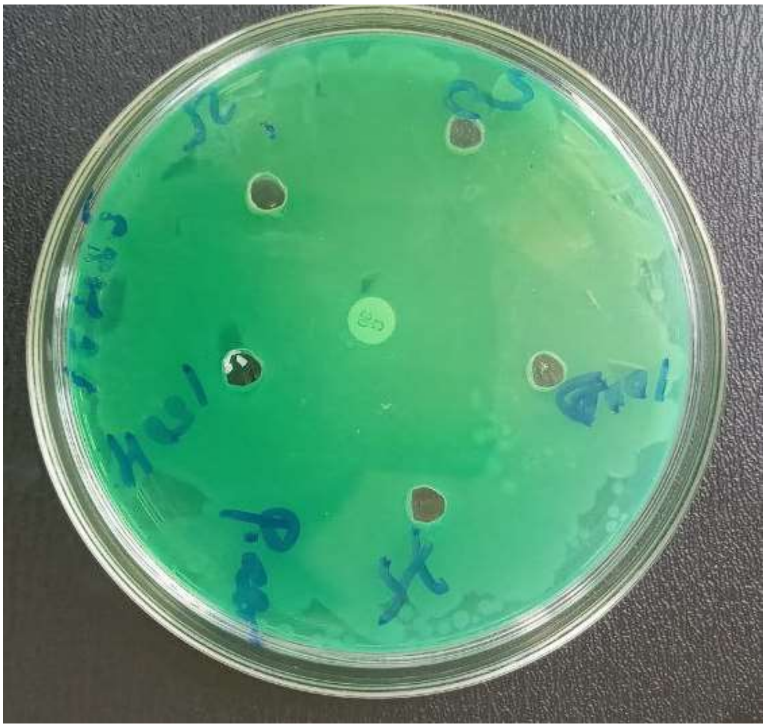

Supplement: Supplementary file 1 — Figure 1: Honey sample taken from beekepeers. Figure 2: Honey sample filtration. Figure 3: Honey samples for LAB analysis. Figure 4: Honey sample pH measurement. Figure 5: Test tube slants of bacterial pathogen. Figure 6: Different honey concentration inoculated on Mueller Hinton agar. Figure 7: Inhibition pattern of honey concentration and sterile water (NC) and PC (Chloramphenicol) against E. coli. Figure 8: Antibacterial activity of honey against P. aeruginosa (No inhibition zone). [file MBO3-14-e70076-s001.pdf]
